# Applications of liposomes and lipid nanoparticles in cancer therapy: current advances and prospects

**DOI:** 10.1186/s40164-025-00602-1

**Published:** 2025-01-31

**Authors:** Zhe Cheng, Huichao Huang, Meilong Yin, Huaizheng Liu

**Affiliations:** 1 Department of Emergency, The Third Xiangya Hospital, Central South University, Changsha, 410013 China; 2 Department of Oncology, NHC Key Laboratory of Cancer Proteomics, Laboratory of Structural Biology, Xiangya Hospital, Central South University, Changsha, 410008 China; 3 Department of Infectious Disease, XiangYa Hospital, Central South University, Changsha, 410008 China

**Keywords:** Liposome, Lipid nanoparticle, Cancer therapy, Targeted delivery, TME, Tumor vaccination

## Abstract

Liposomes and lipid nanoparticles are common lipid-based drug delivery systems and play important roles in cancer treatment and vaccine manufacture. Although significant progress has been made with these lipid-based nanocarriers in recent years, efficient clinical translation of active targeted liposomal nanocarriers remains extremely challenging. In this review, we focus on targeted liposomes, stimuli-responsive strategy and combined therapy in cancer treatment. We also summarize advances of liposome and lipid nanoparticle applications in nucleic acid delivery and tumor vaccination. In addition, we discuss limitations and challenges in the clinical translation of these lipid nanomaterials and make recommendations for the future research in cancer therapy.

**Graphical Abstract:**

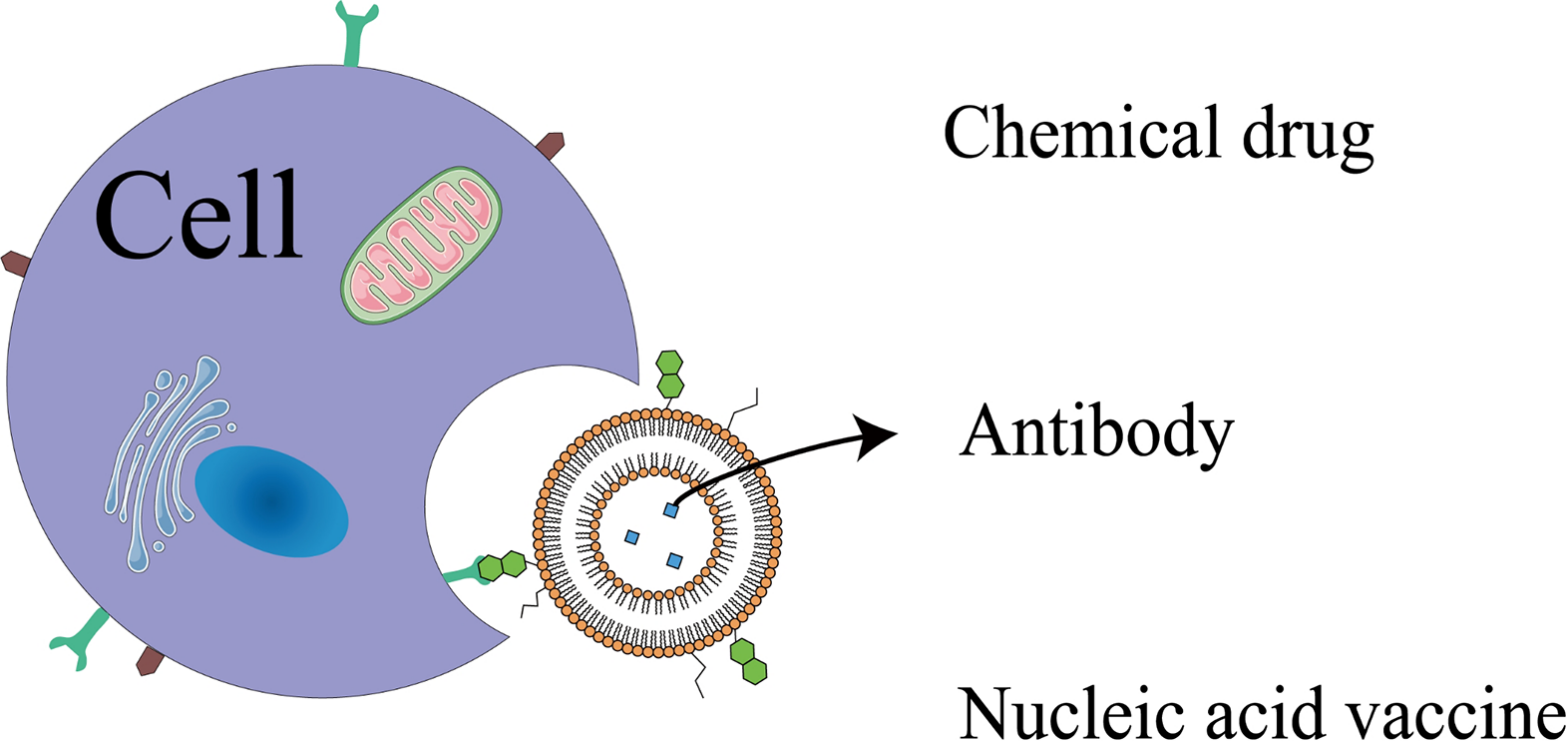

## Background

Cancer is a disease that seriously threatens human health. In 2020, there were estimated over 19 million new cancer cases and nearly 10 million cancer deaths [1]. Cancer is one of the main causes for morbidity and mortality. So far, research has revealed that complex pathological process is involved in cancer occurrence, metastasis and reoccurrence. Genomic instability and mutation are common patterns in tumorigenesis while cell death resistance, induced angiogenesis, inflammation also play important roles in cancer development [2]. Treatment of cancer has always been a major spotlight in medical research. Common cancer treatment methods include surgery, chemotherapy, radiation therapy, targeted therapy, immunotherapy, hormone therapy. As a common approach vastly applied in clinical practice, chemotherapy has been proved to be effective in cancer treatment. To achieve better treatment efficacy, chemotherapy is also combined with surgery [3], radiation therapy and other therapies [4]. However, due to intrinsic characteristics of chemical drugs, all kinds of side effects can be caused.

Chemotherapy inhibits rapid-growing normal cells due to indiscriminate cytotoxicity of chemical drugs. These cells include bone marrow cells, hair follicles, gastrointestinal tract cells and cells in reproductive system. Chemotherapy can also cause multi-drug resistance (MDR) because of distribution heterogeneity and non-specificity of chemical drugs [5, 6]. These side effects greatly affected the treatment outcome. In addition to chemical drugs, certain antibodies and nucleic ingredient also exhibit inspiring anti-tumor efficacy. To prolong the half-life of these therapeutic drugs and specifically deliver them to the target tumor cells, nanocarriers are studies in depth. And lipid-based drug delivery system (LBDDS) is one of the most promising nanoplatforms being researched.

To reduce chemical drug cytotoxicity towards normal tissues and cells, lipid-based nanocarriers are applied in cancer chemotherapy. Nanocarriers used in cancer therapy are generally on the scale of 1–100 nm, and lipid-based nanocarriers are vastly studied in cancer therapy. Lipid-based nanocarriers are nano-scale materials made of lipids. Liposomes, solid lipid nanoparticles (sLNP), nanostructured lipid carriers (NLC), nanoemulsions, nanosuspensions, and niosomes are currently studied lipid-based nanocarriers in medical fields. These lipid-based nanocarriers are researched to deliver drugs in various diseases including skin diseases [7, 8], tuberculosis [9, 10], Alzheimer’s disease [11], infection [12], ophthalmic diseases [13], cardiovascular diseases [14]. lipid-based nanomaterials are also used as key vehicles in mRNA vaccines. Nanoemulsions are colloidal nanoparticles. Compared with liposomes, nanoemulsions require high temperature and pressure, making it cost more in commercial manufacture. Due to lack of understanding of chemistry in NE production, the safety should be well assessed before clinical use [15].

In the fight against coronavirus disease 2019 (COVID-19), lipid nanoparticles constitute mRNA vaccine platform [16, 17]. One of the most extensively applied area of lipid-based nanocarrier is cancer therapy. Once loaded into a lipid-based drug delivery system, chemical drugs, especially those with poor water-solubility, gain better specificity, longer half-life, improved bioavailability. Compared with plain drug solutions, drugs loaded in LBDDSs also gain better pharmacokinetics [18]. With sophisticated design, controllable drug release and variable targeting capability can be achieved [19].

Common LBDDSs such as liposomes and LNPs are used to improve active pharmaceutical ingredient (API) delivery in cancer treatment. In recent years, various active targeting strategies based on antigen-antibody reactions and ligand-receptor binding have been explored in the laboratory, but few can be put into clinical use [20–22]. We summarize advances in liposomal active targeting delivery strategies and bring up possible solutions of obstacles in clinical translation. Nucleic acids are vastly researched in cancer treatment and vaccine development. To achieve better specificity towards target cancer cells, liposome and LNP vehicles used for nucleic acid delivery in cancer treatment can be modified with ligands or moieties. Cancer vaccines encapsulated with nucleic acids are also promising approaches for cancer immunotherapy and numerous researches on mRNA-based cancer vaccines are being carried out.

## The application of common LBDDSs in cancer therapy

There are all kinds of lipid-based drug delivery system (LBDDSs) applied in cancer therapy: Liposomes, LNPs, NLCs, nanoemulsions, nanosuspensions, and niosomes. Although these materials all consist of lipid ingredients, each of them possesses unique structure and heterogeneous characteristics. Liposomes and LNPs are the most common lipid-based nanomaterials based on amount in research and clinical applications. Liposomes have a long history and were first defined in the mid-60s. They are usually described as spheres consisting of single or multiple phospholipid bilayers [23]. Size and structure are two major parameters that affect characteristics of liposomes. Liposomes with unilamellar bilayer lipid membrane can be divided into small unilamellar vesicles (SUV) with size within 100 nm and large unilamellar vesicles (LUV) with size over 100 nm [24]. Multilamellar vesicles (MLV) are liposomes with more than one bilayer lipid membrane. Liposomes can also encapsule several smaller micron-sized non-concentric liposomes, in which case multivesicular liposomes (MVL) are manufactured. MVLs can provide sustained drug release when applied in non-vascular administration due to this particular structure [25]. Liposomes are composed of sterols, surfactants and natural or synthetic phospholipids, such as egg yolk, soybean and hydrogenated phosphatidylcholine [26, 27]. These ingredients are biodegradable, non-toxic and suitable for industrial production. The hydrophilic core and hydrophobic phospholipid bilayer make it possible to encapsule both hydrophobic and hydrophilic drugs. Compared to free cancer chemical drugs, liposomes help reduce undesired toxicity towards normal cells and tissues, prolong half-life, enhance drug solubility, improve drug pharmacokinetics and improve drug bioavailability.

Liposomes are broadly used in food, medicine, cosmetics and other areas [28, 29]. Liposomes, nanoparticles (NPs) and micelles are vastly used nanomaterials in clinical medicine [30, 31]. In cancer treatment, liposomes have been utilized as ideal DDSs to deliver chemical drugs. There are two factors to attend to when designing liposomal DDSs for cancer treatment: encapsuled drugs/therapeutic substances and surface modification. These nano-DDSs are phagocytized by mononuclear phagocyte system (MPS) in liver and spleen, to further prolonged drug half-life, improve drug solubility and stability, reduce drug immunogenicity and antigenicity, polyethylene glycol (PEG) is used to modify liposome surface. PEGylated liposomes are harder to be recognized by MPS therefore are delivered to cancerous areas more efficiently. To date, several liposomal DDSs have been approved in cancer treatment, and some are PEGylated. These liposomal designs have shown concrete therapeutic effects in cancer treatment. Moreover, liposomes are modified to achieve better efficiency in cancer therapeutic material delivery.

**Fig. 1:**
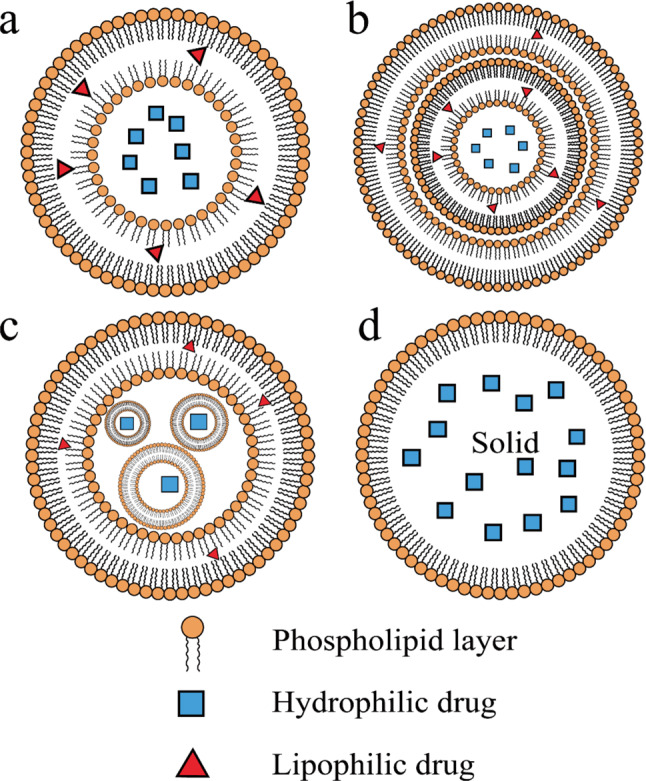
Structures of various liposomes and LNPs. **a** SUVs (within 100 nm) and LUVs (over 100 nm). **b** MLVs. **c** MVLs. **d** LNPs. LNP: Lipid nanoparticle; SUV: Small unilamellar vesicles; LUV: Large unilamellar vesicles; MLV: Multilamellar vesicles; MVL: Multivesicular liposomes

LNPs and NLCs are LBDDSs that differ in composition and structure from liposomes. LNPs are colloidal nanocarriers among 1–100 nm, and LNPs are solid particles at body temperature. NLCs consist both solid and liquid lipids. Compared with traditional liposomes, these recently developed lipid nanomaterials have prolonged release and relative higher drug stability. However, solid composition also causes unpredictable gelation tendency and leads to insufficient incorporation rates [32]. LNPs and metallic nanoparticles are both nano-scale particles with diameter within 1000 nm. This similarity indicates they can be vehicles transporting therapeutical ingredients to tumor cells [33, 34]. Unlike metallic NPs, LNPs are more easily cleared from the biological system and causes less accumulation in tissues, therefore LNPs are considered to exhibit less toxicity accumulation in the organism [35, 36]. Structures of various liposomes and LNPs are shown in Fig. 1.

Nanoemulsions, nanosuspensions and niosomes are also explored in cancer therapy [37–40]. Doxil is the first liposomal nano-DDS approved by FDA (Food and Drug Administration). Doxil is a liposomal DDS that encapsulates doxorubicin, it is PEGylated in the surface and used in various cancer treatment including breast, bone, ovarian, breast, lung, brain cancer, leukemia and AIDS-related Kaposi’s sarcoma. Doxil has achieved concrete clinical success since it was developed and approved by FDA in 1995 [41]. To date, almost all FDA-approved LBDDSs have been manufactured based on liposomes [42], suggesting that liposomes are one of the most promising lipids nanoplatforms (Table 1). Lipid-based nanocarriers using various therapeutic approaches including chemotherapy, immunotherapy and nucleic acid drugs have also been tested by clinical trials (Table 2) (https://clinicaltrials.gov/).

**Table 1:** Summary of FDA-approved LBDDSs in cancer therapy

Brand name	Pharmaceutical ingredients	Indication(s)	Composition	Approval year
Doxil	Doxorubicin (DOX)	Breast, bone, ovarian, breast, lung, brain cancer, leukemia, AIDS-related Kaposi’s sarcoma	HSPC, Cholesterol, DSPE-PEG 2000	1995
DaunoXome	Daunorubicin	Advanced HIV-associated Kaposi’s sarcoma	DSPC, Cholesterol	1996
Depocyt	Cytarabine	Lymphomatous meningitis	DOPC, DPPG, Cholesterol, Triolein	1999
Marqibo	Vincristine	Acute Lymphoblastic Leukemia	Sphingomyelin, Cholesterol	2012
Onivyde	Irinotecan	Metastatic pancreatic cancer	DSPC, MPEG-2000, DSPE	2015
Vyxeos	Daunorubicin and cytarabine	Acute myeloid leukaemia	DSPC, DSPG, Cholesterol	2017

**Table 2:** Examples of cancer LBDDSs in clinical trials (as of January 2025)

Therapy	Nano-platform name	Indication(s)	Composition	NCT number	Status	Outcome (year)
Chemotherapy	EndTAG-1(2016)	Breast cancer	Liposomal paclitaxel	NCT03002103	Phase III trial ongoing Suspended	Undisclosed (2024)
Chemotherapy	MM-302(2014)	Breast cancer	HER2-targeted liposomal doxorubicin hydrochloride	NCT02213744	Phase III trial terminated	Negative (2017)
Chemotherapy	ThermoDox(2009)	Breast cancer	Thermally sensitive liposomal doxorubicin	NCT00826085	Phase III trial completed	Positive (2017)
Immunotherapy	DPX-Survivac(2014)	Lymphoma	Liposomal survivin-based synthetic peptide antigens	NCT02323230	Phase II trial terminated	Negative (2021)
Immunotherapy	PNT2258(2012)	Lymphoma	Liposomal DNA interference oligonucleotides PNT100	NCT01733238	Phase II trial completed	Positive (2020)
Nucleic acid	SGT-53(2015)	Pancreatic cancer	Liposomal p53 plasmid	NCT02340117	Phase II trial recruiting	Recruiting
Nucleic acid	TKM-PLK1(2010)	Neuroendocrine tumors and adrenocortical carcinoma	Lipid NP containing PLK1 siRNA	NCT01262235	Phase II trial completed	Undisclosed (2015)
Nucleic acid	WGI-0301(2022)	Advanced solid tumors	Lipid NP Suspension of Akt-1 antisense oligonucleotide	NCT05267899	Phase I trial recruiting	Recruiting

LNPs are colloidal particles with the ability of nucleic acid encapsulation and delivery. Genetic drugs such as mRNA, small interfering RNA (siRNA) and plasmid DNA are used in gene therapy [43]. LNPs were originally designed to deliver siRNAs, and recently LNPs were used in mRNA delivery in cancer gene therapy and cancer vaccine development [44, 45]. Encapsulation of these therapeutic nucleic acid helps improve their stability, facilitate internalization, limit immune activation and increase target specificity. We summarize recent advances of LNP application in cancer gene therapy and cancer vaccine development.

## Liposomes and targeted delivery

Conventional chemical drugs are used in cancer chemotherapy. Because of indiscriminate cytotoxicity towards fast-growing cells, these free drugs exhibit various side effects. Nanomaterial based DDSs are applied to reduce these undesired side effects. Once loaded into liposomes, chemical drugs as well as other therapeutic materials gain better specificity towards cancer cells. In liposomal DDS targeting area, two major techniques are used: passive targeting and active targeting.

### Passive targeting

Passive targeting is primarily based on the enhanced permeability and retention (EPR) effect created by the specific structure of the cancer tissue. In comparison to normal tissue, cancer tissue forms plentiful aberrant vascular and excessive vascular permeability factors, and lack of lymphatic drainage. These features help liposomal DDSs target at cancer cells and achieve enrichment at cancerous areas. With better specificity, liposome encapsuled chemical drugs show less toxicity to normal organs or tissues, therefore lead to fewer side effects [46]. Although EPR effect has been reported and studied broadly, most nanodrugs based on EPR effect show unfavorable targeting efficacy. The reason is that EPR effect has its limitations. Research showed that the frequency of gaps in tumor tissues could not fully explain nanoparticle accumulation in tumor. Further research showed that EPR effect possesses quite heterogeneity across tumor types and patient individuals [47, 48].

**Fig. 2:**
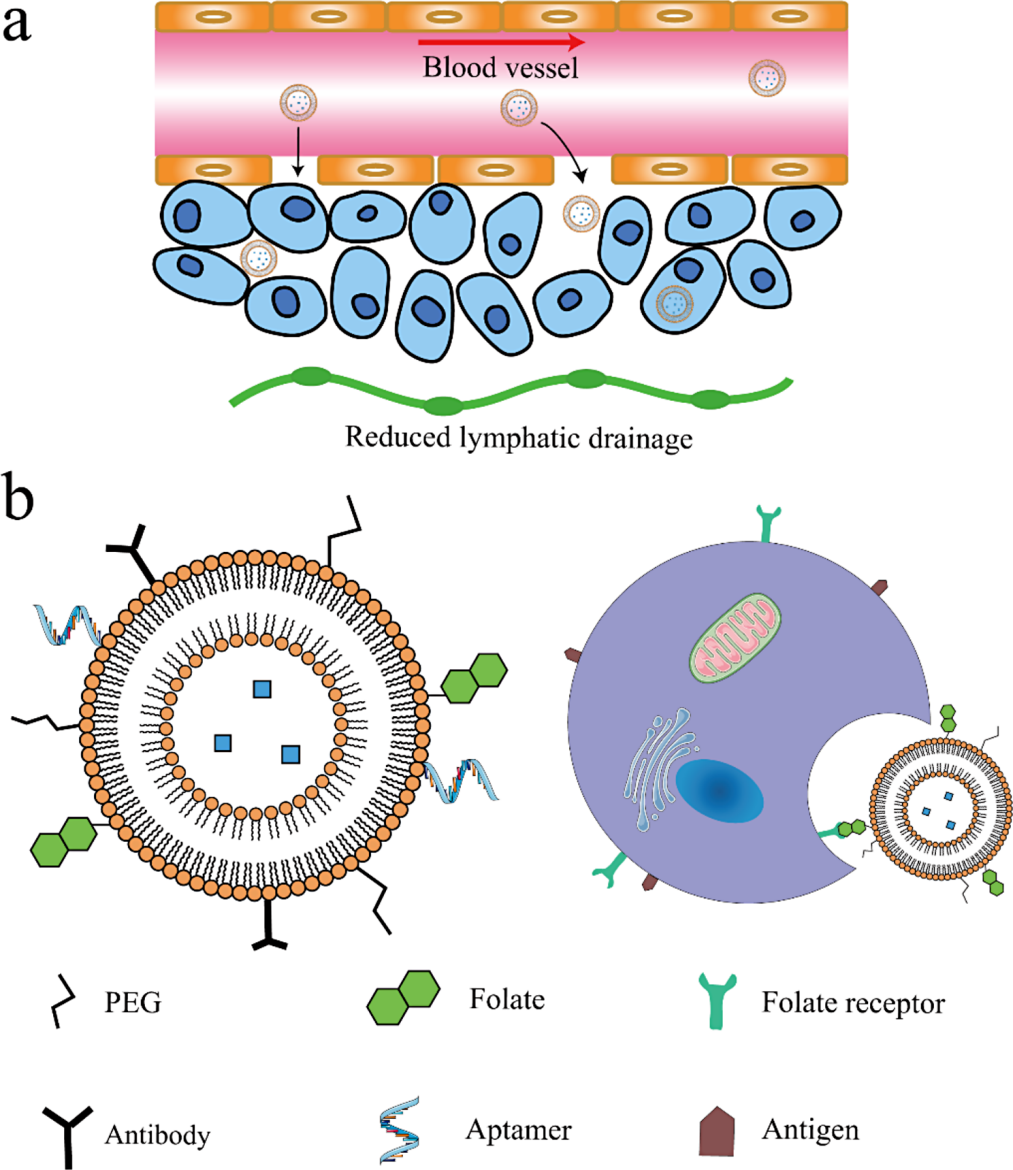
Schematic representation of passive and active targeted delivery. **a** Liposomal nanocarrier delivers drugs to cancer cells through EPR effect. **b** Illustration of liposomal active targeted delivery strategies. Liposome endocytosis and transcytosis mediated by folate-FR conjugation

Liposomal DDS are nanomaterials, therefore possess basic characteristics such as high surface-to-volume ratio, enhanced biocompatibility and increased permeability through biological barriers [49]. However, compared with other nanomaterials such as polymeric NPs, metallic NPs, intrinsic electrical conductivity, superparamagnetic behavior or optical features of liposomes are less used in medical fields. Over the years, active targeted liposomal DDS have been vastly researched. Active targeting is commonly realized by conjugating certain ligands to liposome surface. These modified liposomes mainly target at two categories: cancer cells and tumor microenvironment (TME). TME refers to the environment setting that promotes tumor. TME includes fibroblasts, endothelial cells that associated with cancer, immune cells and extracellular matrix also interact with cancer cells [50]. Research show that innate and adaptive immune cells in TME can secrete growth factors and cytokines that contribute to tumor invasion, metastasis, interactions of cancer cells and TME immune cells also attenuate antitumor immunity [51, 52]. Surface of liposomal DDSs are modified with various molecular moieties such as antibodies, proteins, peptides, aptamers. Most of these moieties bond specifically to receptors that are highly expressed in cancer cells or TME, while some bond to enzymes that contribute to tumor growth. It should be noted that the active targeting strategy that conjugates liposome with molecular moieties is based on the EPR effect, and the moieties contribute to liposome internalization process (Fig. 2).

### Active targeting strategy

#### Ligands

Ligands are commonly used in active targeting in liposome manufacture. As ligands bind to corresponding receptors, specific ligands help guide the liposomal nano-DDS to enrich in target cancerous areas. Transferrin receptor (TfR), folate receptor (FR), estrogen receptor (ER) and hyaluronic acid receptor are vastly investigated ligand-binding targets. TfRs are glycoproteins that bind to iron in blood plasma. TfR plays important roles in cellular iron uptake. In normal cells, TfR is highly expressed on placental tissue, immature erythroid cells and other rapidly dividing cells [53]. Tumor cells are rapidly proliferating cells and TfR is highly expressed in many cancer cells. Researches show that TfR is heavily distributed in blood-brain barrier (BBB) and glioblastoma multiforme (GBM) cells [54, 55]. TfR is also found to be highly expressed in other solid tumors such as breast cancer and hepatocellular carcinoma. Research indicates that TfR1 overexpression can be a potential prognostic indicator of hepatocellular carcinoma [56]. Zhang et al. developed a doxorubicin (Dox)-loaded liposomal nanocarrier, and the holo-lactoferrin (holo-Lf) was attached to the nano-DDS as it is a natural protein that acts as a ligand of TfR. The nanocarrier exhibited satisfactory cellular uptake [20]. Compared to single target modified liposomes, some liposomes are co-modified with both TfR and penetration enhancers. These designs accomplish better specificity and endocytosis efficacy towards target cells. Desphande and coworkers developed a dual-functional liposome modified with both transferrin and octaarginine, a popular penetration enhancer by improving cell micropinocytosis. The liposomal nanocarrier loaded with Dox showed improved drug accumulation in ovary tumors [57].

Folate receptor is also a common target in active targeting strategies. Folate is converted from folic acid in the body. Folate plays important roles in nucleotide base synthesis and cell division. There are several isoforms of FRs and some FRs are expressed on the surface of epithelial cells [58]. FRs are found to be overexpressed in many tumors including liver cancer, lung cancer, breast cancer, ovarian cancer, prostate cancer [59–62]. In liposomal nano-DDS design, FRs are often used as practical targets to guide the nanocarrier. As folate receptor is overexpressed in lung cancer cells, many researches have conjugated folate onto the nanocarrier to gain better specificity towards lung cancer cells. Park et al. developed a pH-sensitive liposome carried with doxycycline and docetaxel in non-small cell lung cancer (NSCLC) treatment. The liposome was modified with folate in the surface to specifically target at M2 tumor-associated macrophages and NSCLC cells, and the liposome exhibited specific targeting ability and controlled release ability [63]. A topotecan liposome was modified with folate and exhibited improved therapeutic efficacy against A549 lung cancer cells [64]. A delicate liposome-based nano-DDS was developed by Lv and coworkers, the liposome platform was modified with folate and encapsulated with magnetic nanoparticles, gold nanorods and DOX, and the liposomal DDS could specifically bind bladder tumor cells and exert cytotoxicity [65].

Another vastly studied target in active target liposome design is estrogen receptor. ER binds to estrogen that play important roles in women’s sexual and reproductive system. There are two subtypes of ER: Erα and Erβ. ER expression varies in human cancers. There are approximately 75% Erα positive breast cancer cases at diagnosis [66]. ER is used as a potential target in ER-positive cancer treatment including breast cancer, gastric cancer and acute myeloblastic leukemia [67, 68]. Estrone targeted liposomal nano-DDS have been developed to achieve better drug efficacy. Xu et al. [69] developed an estrone-targeted liposomal system to deliver mitoxantrone in human acute myeloblastic leukemia treatment, and the in vivo experiment conducted in mouse showed that the liposomal nano-DDS could be well delivered to targeted ER-expressing tumor cells. Drug circulation time was prolonged and drug side effects was reduced. Chemotherapy is the main option in advanced metastatic gastric cancer treatment [70]. However, oxaliplatin, the chemo-drug used results in serious side effects including nausea, vomiting, mucosal inflammation and neurological toxicity [71]. An oxaliplatin loaded, estrogen targeted PEGylated liposome was developed and exhibited concrete anti-tumor efficacy, improved metabolic behavior and reduced toxicity towards normal tissues [72]. ER is also used as practical target in cervical cancer nano-DDS design. Li and coworkers designed a PEGylated liposomal nano-DDS platform for cisplatin delivery. The target specificity and metabolic behavior was improved and myelosuppression was decreased [73].

Cluster of differentiation 44 (CD44) is a glycoprotein expressed on cancer cell surface. CD44 overexpress in many tumors including breast, pancreatic, ovary, brain and lung tumors [74]. CD44 specifically binds to hyaluronic acid (HA) and chondroitin sulfate (CS), therefore can be utilized as a target tool in liposomal platform design. CD44 was found to be related to various physiological and pathological functions including angiogenesis, cell differentiation, proliferation and tumor metastasis [75]. A stimuli-responsive polysaccharide modified liposomal platform with CD44 targeted ability was developed. The redox-sensitive amphiphilic CS and HA were conjugated onto the liposome and therapeutic shRNA that silences the inhibitor of apoptosis was loaded into the liposome. The liposomal carrier suppressed the tumor growth and exhibited low toxicity to mice [76]. Dong et al. developed an epalrestat and DOX dual-loaded liposome in triple-negative breast cancer treatment, and HA tagged on the surface provided effective target ability through CD44-HA interaction. The liposome suppressed tumor growth and metastasis [77]. These ligand-receptor active targeting strategies are closed associated with receptor expression level in target cancer cells, and with specific ingredients that enhance permeability and bioavailability, ligand-based liposomal DDS exhibit satisfactory delivery efficacy.

#### Proteins and peptides

The specific antigen-antibody conjugation has been utilized to achieve better targeted ability of liposomes. Since various proteins are expressed on cancer cell and TME cell surface, antibodies of these proteins can be used to help target at desired cancerous sites. Once conjugated with corresponding antibodies, receptors on cell surface can also cause series of changes of downstream signaling and exert anti-tumor effect. Therefore, active targeting strategy can be closely related with targeted therapy that aims at specific targets in cancer targeted therapy. Epidermal growth factor receptor (EGFR) is an important signaling component that regulates various processes such as cell proliferation, differentiation, growth and inhibition of apoptosis [78]. Combination with epidermal growth factor (EGF) results in downstream signal transduction, which are PI3K/AKT/mTOR, Ras/MAPK and PLC/PKC signaling or changes in expression level [78, 79]. PI3K/AKT/mTOR signaling pathway regulates cell metabolism, proliferation and cell survival. This pathway is often heavily activated as a result of EGFR mutation. LBDDSs can be designed to specifically target at EGFR and inhibit it, which leads to inhibition of PI3K/AKT/mTOR activation. Since activation of EGFR regulates signaling network and affect cancer cell proliferation, migration and apoptosis, EGFR and related proteins have become effective targets for cancer treatment. EGF can be conjugated with liposomes to enhance targeted ability of liposomal nano-carrier. Silver nanoparticles (AgNPs) are toxic therapeutic materials for both cancer and healthy cells, and to reduce toxicity towards normal cells, researchers developed an EGF-labeled AgNP loaded liposome. The liposomal nano-platform exhibited fewer toxicity against normal human fibroblasts and elevated proapoptotic effect and toxicity against EGFR-overexpressing cancer cells [80]. In pancreatic cancer treatment, EGF is also used to enhance the targeted ability of liposomes. Le and coworkers fabricated an EGF-conjugated liposome containing curcumin. The liposomal nanocarrier was tested with human pancreatic cancer cell lines that express EGFR, and the EGF-decorated liposome enhanced the antitumor activity of curcumin in pancreatic cancer chemotherapy [21]. Other than ligand-receptor combination similar to EGF-EGFR, antibodies of target receptors can also be used to modify liposome surfaces and used in targeted delivery. With these specific antibodies, the liposomal platform can combine chemotherapy and targeted therapy at the same time. Cetuximab is an anti-EGFR monoclonal antibody. DTX is broadly used in metastatic prostate cancer chemotherapy. Eloy et al. designed a targeted immunoliposome with cetuximab modified on the surface. And the cetuximab targeted liposome had high DTX encapsulation efficiency. Higher cellular uptake and toxicity in EGFR-overexpressing prostate cancer cells DU145 was exhibited compared to non-targeted liposomes [81]. Another member of human epidermal growth factor receptor family is human epidermal growth factor receptor 2 (HER2), which is also a transmembrane glycoprotein that possesses tyrosine kinase activity. Although HER2 doesn’t bind any ligand, it is essential in mediation of signaling pathways [82]. HER2 is an important target for cancer treatment as HER2 overexpresses in many cancers and is found to be strongly associated with increased recurrence and poor prognosis [83]. HER2 antibody can also be utilized in liposome surface modification. Li and coworkers built a light-sensitive liposome consisting of hydrophobically modified photosensitizer (ICG-ODA) and special phospholipid (PLsPC). DOX was encapsuled to exert anti-tumor effect while the surface was modified with HER2 antibodies to target specifically at high HER2 receptor expression tumor cells. The liposomal system showed significant accumulation in MCF7 and SKOV3 tumor cells due to mediation of HER2 antibody [84]. As TfR is heavily expressed in BBB and GBM cells, TfR can also be used in active targeting strategy. A docetaxel (DTX) liposome co-modified with muscone/RI7217 was developed to treat glioma. RI7217, a mouse monoclonal antibody that possesses high affinity and selectivity for TfR, was modified in the surface of the liposome. Muscone was reported to inhibit P-glycoprotein and matrix metalloproteinase-9 expression and thereby relax the epithelial cell junction. Muscone alters BBB permeability and boosts drug uptake in the brain. The liposomal DDS exhibited improved brain targeting efficacy [85].

In active targeted liposomal manufacture, antibody fragments (Fab) and single-chain variable fragment (scFv) are also used in liposomal surface modification [86]. Because antibody may contain more than one reactive group, heterogeneity can be generated when the antibody is conjugated on the liposome surface, and improper orientation towards the antigen also leads to targeted efficacy heterogeneity [87, 88]. And research show that covalent reactions that bind antibody to liposome might potentially affect the antibody or the liposome, leading to heterogeneity in targeting efficacy [89]. Therefore, various modification methods have been explored to modify antibody or their peptide fragment. A recombinant Fab antibody fragment was generated, and the protein D derived from pulmonary surfactant was fused on the C-terminus of the fragment. The fused protein D can be inserted into liposome bilayer and anchored the Fab to the liposomal surface [86]. Li and coworkers fabricated two immunoliposomes conjugated with anti-EGFR-Fab’, and siRNA was encapsuled as therapeutic agents. The liposome-polycation-DNA complex (LPD) exhibited good targeting and gene silencing ability [22]. Compared with the whole antibody molecule, the Fab/scFv used in these researches exhibit reduced immunogenicity and improved pharmacokinetics. Peptide fragments also have easier preparation, lower cost, decreased opsonization, lower antigenicity and better stability against enzymatic degradation [90]. These properties make peptide more beneficial than whole antibodies in clinical use.

Targeting TME is also an important active targeting strategy. Blood vessels, fibroblast, signaling molecules, extracellular matrix (ECM) and immune cells in TME are possible targets in anti-tumor liposome design [91–93]. Immune checkpoint molecule is an important category in cancer immune. Regulators of immune responses can also become targets for active targeting strategies. Programmed cell death ligand 1 (PD-L1) is known as an immune checkpoint that overexpresses on tumor cells. It binds to programmed death-1 (PD-1) receptor present on activated effector T cells [94]. PD-1/PD-L1 interaction down-regulates T cell antitumor activity and reduces inhibitory cytokine secretion, therefore can suppress immune function and promote tumor progression [95]. Liposomes targeting on PD-1/PD-L1 axis are developed based on these researches. A dual activity immunoliposome loaded with DOX was developed and tested in melanoma murine model. A monovalent-variable fragment (Fab’) of PD-L1 monoclonal antibody was modified onto the liposome surface. Result showed that the immune checkpoint inhibitor (ICI) modified immunoliposome bound specifically to PD-L1 + cells and decreased 30-fold the IC50 compared to conventional liposomes [96]. There are massive researches towards TME and receptors related to cancerous angiogenesis, vascular co-option, cell adhesion, cell migration are studies as targets for liposome design [97, 98]. Vascular endothelial growth factor (VEGF) is a vital regulator in cancer abnormal angiogenesis and the vascular endothelial growth factor receptor-2 (VEGFR-2) overexpresses in human breast cancer cells. A DTX encapsuled functional VEGF antibody modified liposome was fabricated in breast cancer treatment. The result showed that cellular uptake of DTX carried by liposome was significantly higher than free DTX and the liposomal nanocarrier resulted in significant reduction in tumor burden [99]. Researches aiming at TME remodeling and immune regulation through liposomal delivery have also been vastly conducted [100–102].

#### Aptamers

Aptamers are short single-stranded DNA (ssDNA) or single-stranded RNA (ssRNA). The in vitro approach called systematic evolution of ligands by exponential enrichment (SELEX) is a major approach to isolate evolution of synthetic ssDNA/ssRNA during aptamer production in vitro [103, 104]. Similar to antibodies, aptamers can also specifically bind to corresponding targets with high affinity [105, 106]. However, compared to antibodies, aptamers bear better properties including higher thermal stability, less immunogenicity. And lower production cost of aptamers allows large-scale synthesis [107, 108]. Aptamers possess better tumor penetration and retention properties because of smaller size. Homogenous distribution also makes it ideal for modification of targeted delivery liposomes [109]. Aptamers can be conjugated to surface of nanomaterials with higher density and less steric hindrance compared to antibodies fragments. To date, novel liposomal drug carriers modified with aptamers have been fabricated and tested in cancer therapy. Active pharmaceutical ingredient (API) is the key component of liposomal DDS. BRAF gene mutation is common in over 60% malignant melanomas. An active targeted liposome encapsuled with anti-BRAF siRNA (siBraf) was developed in melanomas treatment. AS1411 is an aptamer specifically binds to nucleolin and the PEGylated cationic liposome was modified with AS1411. The AS1411-PEG-liposome exhibited significant silencing activity of BRAF gene and higher siRNA accumulation compared with normal cells. The liposomal nanocarrier inhibited the melanoma growth [110]. AS1411 was also utilized in other aptamer-functionalized liposomes in breast cancer and basal cell carcinoma treatment [111, 112]. These studies suggest that aptamers have great potential in active targeted liposome design. To sum up, various materials have been utilized in active targeting strategies and achieved favorable results (Table 3). In addition to employ various moieties to achieve active targeting, stimuli-responsive strategies can also enhance the targeting efficacy of the nano-drug.

**Table 3:** Active targeted liposomes used in cancer therapy

Targeted moieties	API	Target cancer model	Anti-cancer effect	References
Holo-lactoferrin (holo-Lf)	Dox	Murine breast cancer cell line 4T1	The Lf-liposome-Dox relieved tumor hypoxic microenvironment and achieved excellent cancer treatment effect combined with radiotherapy.	[20]
Antibody RI7217	DTX	U87-MG glioma cells and immortalized hCMEC/D3	The muscone and RI7217 modified DTX liposome showed improved brain targeting and prolonged survival time of tumor-bearing mice.	[85]
Folate	Doxycycline and docetaxel	Human NSCLC cell line A549	Effectively inhibited tumor growth and reduced drug side effects.	[63]
Estrone	Mitoxantrone	Human acute myeloblastic leukaemia HL-60 cells	The liposomal nanocarrier effectively entered into ER-expressing cells and accumulated, prolonged circulation time and reduced side effects.	[69]
EGF	Curcumin	Human pancreatic cancer cell lines, BxPC-3, Panc-1 and Mia Paca-2	The curcumin loaded liposome exhibited enhanced antitumor activity with surface modified EGF.	[21]
Anti-EGFR-Fab’	siRNA	Human hepatocellular carcinoma cell line SMMC-7721	Immunoliposomes conjugated with anti-EGFR Fab’ showed higher luciferase gene silencing efficiency and were more effective in targeting hepatocellular carcinoma cells for siRNA delivery.	[22]
VEGF antibody	DTX	Human breast cancer cell line MCF-7	The VEGF modified liposome exhibited enhanced cellular uptake and cytotoxicity.	[99]
AS1411	siBraf	Melanoma cell A375	The AS1411 modified liposome showed excellent tumor-targeting ability, silencing activity and melanoma inhibition in A375 tumor xenograft mice.	[110]

## Stimuli-responsive strategy and combined therapy

**Fig. 3:**
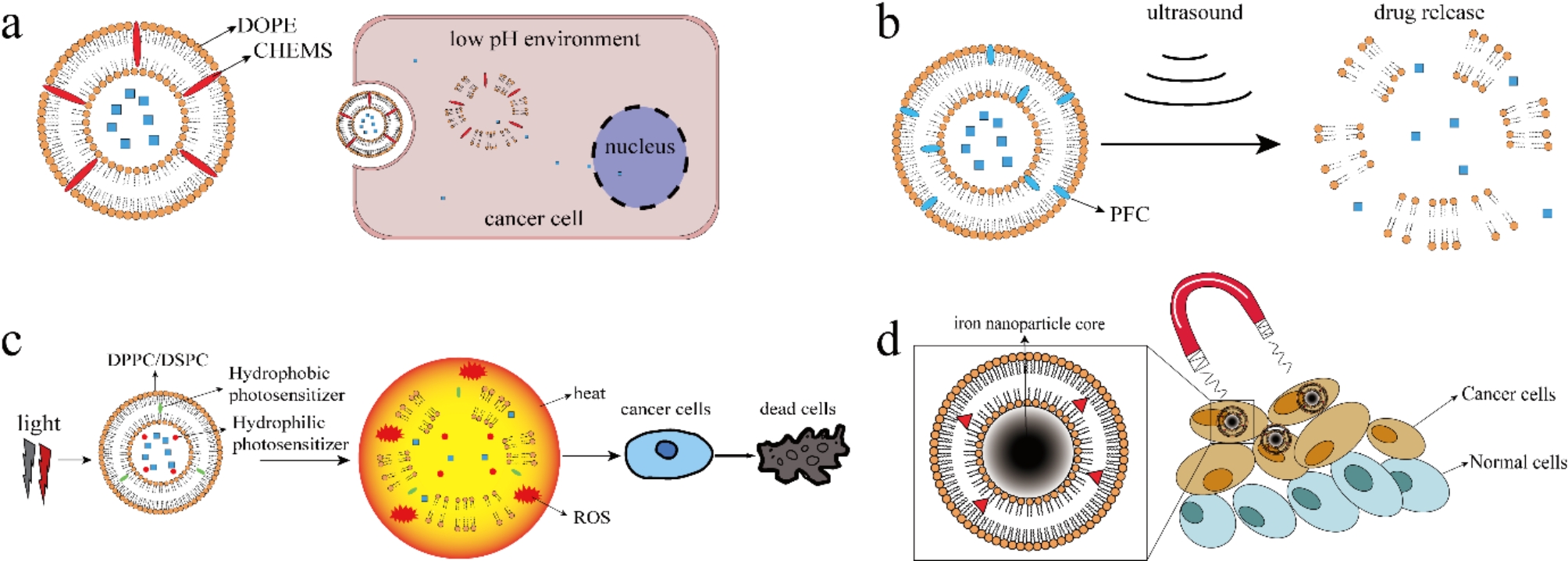
Stimuli-responsive strategies applied in targeted delivery. **a** pH-sensitive liposomes release anti-tumor drugs in low pH cancer cell environment. After entering the acid environment in cancer cells, the DOPE disassembles and releases the ingredients. **b** Ultrasound-sensitive liposomes release anti-tumor drugs under ultrasound stimulate. **c** Illustration of light-sensitive liposome induced PTT and PDT therapy. The lipid was resolved upon light stimulate and release the drug cargo. **d** Schematic representation of magnetic guided liposomes encapsulated with nanoparticle core. The nanocarrier was attracted by the magnetic field and enriched in cancerous sites

These proteinic and nucleic acid fragment provide specific active binding of modified liposomes and cancerous target based on EPR effect. Another technique studied in liposomal targeted delivery is stimuli-responsive strategy. This method is based on characteristics of TME and unique properties of modified liposomes (Fig. 3). Acid environment allows possibility of pH-responsive liposomes. As pH level in cancerous tissues is lower than normal tissues, researchers have conducted experiments in pH-sensitive liposome design. Two strategies can be taken in the process: either by aiming at choosing specific composition of the system or anchoring polymers to the liposomal membrane. To make pH-sensitive liposomes, unsaturated phosphatidylethanolamine (PE) can be used. Typical PE includes diacetylenic-phosphatidyl-ethanolamine (DAPE), palmitoyl-oleoyl-phosphatidyl ethanolamine (POPE) and dioleoyl-phosphatidyl-ethanolamine (DOPE) [113]. Mildly acidic amphiphiles such as oleic acid, cholesteryl hemisuccinate (CHEMS) and N-palmitoyl-l-homocysteine (PHC) are combined with DOPE and stabilize the liposome at neutral pH. In acid environment, carboxyl groups of acidic amphiphiles are protonated and eventually result in membrane destabilization of pH-sensitive liposomes and release of encapsulated cargo. Another common approach is to conjugate pH-sensitive peptide/proteins or synthetic polymers to liposomal surface [114]. GALA peptide (WEAALAEALAEALAEHLAEALAEALEALAA), listeriolysin O and the N-terminus of hemagglutinin are available pH-sensitive peptide/proteins. These ligands are inactive in neutral pH environment and undergo conformational changes in lower pH environment, which leads to fusion of liposomal membrane and cell membrane [115]. pH-responsive feature is utilized in liposome design in researches [99]. Zhang et al. used the inorganic nanomaterial MnO_2_ to construct sustained-release liposome. Since MnO_2_ responses to low pH and GSH, the nano-platform exhibited good tumor-specific responses. The liposome consisted of hydrophilic MnO_2_, gefitinib and bevacizumab and was tested in NSCLC treatment. The result showed that the liposomal nano-drug inhibited A549 cell progression with excellent biocompatibility [116].

Oxidative extracellular environment caused by hypoxia in cancerous tissues allows function of redox-responsive liposomal platforms [117]. A novel redox responsive liposomal formulation was manufactured and tested in breast cancer and lung cancer treatment. 7-Ethyl-10-hydroxycamptothecin (SN38) is a camptothecin derivative that targets specifically at DNA topoisomerase I cleavage complexes and has shown great potential in solid tumor treatment. To improve its poor solubility, chemical and metabolic stability, a liposomal nano-DDS was fabricated. SN38 was conjugated with lysophospholipid and release of the parent drug can be triggered by high GSH condition. The liposomal platform showed lower systemic toxicity and improved tumor inhibition [118]. As there are some enzymes exhibit higher expression level in TME compared with normal tissues, enzyme-responsive liposomes can be fabricated with improved target ability. As mentioned before, many enzymes significantly overexpress in cancerous tissues, which is the basis of enzyme-responsive liposomes design. Two main enzymes are vastly researched in this category: matrix metalloproteinase (MMP) and phospholipase enzymes. MMPs are a group of proteases that play important roles in protein degradation in the extracellular matrix. MMPs are closely associated with tumor invasion, metastasis and angiogenesis, and expression level of MMPs is elevated in many cancers. To enable liposome with MMP-responsive ability, MMP-responsive peptides are synthesized and conjugated to the liposome membrane. Once the liposome reaches cancerous sites with high MMP expression, the liposome releases encapsulated API. It was reported that a HER-2 targeted liposome was conjugated with PEG layer. And a protease-sensitive cleavable peptide linker was inserted in the PEG base. MMPs can cleave the PEG and result in substantial oxaliplatin release [119]. Light, ultrasound, heat and magnetism can also be used to design stimuli-responsive liposomes [120]. Conventional cancer chemotherapy causes various undesired side effects, and liposomal nanocarrier reduces these side effects. Besides chemical drugs, RNA, small molecule inhibitors and metallic materials are also common antitumor compound. Combined therapy based on co-delivery of API has been an important approach to achieve higher therapeutic efficacy in liposome research. However, API co-delivery and combined therapy has only been explored in experiments and few has been approved in clinical use. To enhance therapeutic efficacy of the nano-DDS and gain improved side effects, many liposomal platforms are designed with complex compositions and therefore bear multiple features. These novel liposomal vehicles might be conjugated with active targeting ligands, encapsuled with several therapeutic APIs, and unique components enable stimuli-release feature.

Deng and coworkers developed a liposomal spherical nucleic acid encapsulated with DOX and CPG that increases infiltration of immune cells in TME by binding to Toll-like receptor 9 (TLF-9) in the endosome. By incorporating MMP-9-responsive peptide into the lipid nanocarrier, the nanoplatform can response to MMP-9 enzyme in cancer tissues and release therapeutic cargos. The result showed that the MMP-9 responsive-liposome achieved synergistic therapeutic effect by delivering chemical drugs and adjuvants with reduced side effect compared with free drugs [121]. Arsenic trioxide (ATO) is a therapeutic chemical against hepatocellular carcinoma. However, clinical application of ATO has been severely hindered because of its poor distribution and high toxicity [122, 123]. A liposomal drug delivery system was developed to overcome the drawbacks. The nanoplatform consists of arsenic prodrug, MMP2-responsive cleaved cell-penetrating peptide and folate. The calcium arsenate nanoparticle was coated with liposome. The liposomal nanocarrier exhibited better tumor targeting specificity and therapeutic efficacy both in vitro and in vivo [124]. Another target enzyme is phospholipase and subtype secretory phospholipase A2 (sPLA2) is overexpressed up to 22-fold in metastatic prostate cancers [125]. Experiments were conducted using sPLA2 sensitive phospholipids and confirmed the stimuli-responsive feature in cancer treatment [126]. However, there are researches indicate that though sPLA2 responsive liposomes might exhibit anti-tumor effects in vitro, the therapeutic index of sPLA2 triggered release is narrow and may cause severe systemic toxicity in vivo [127–129]. Therefore, stability and toxicity of phospholipase sensitive liposomes should be more carefully evaluated.

For stimuli-responsive strategies, consistent and concrete controlled release is always desired. Unfortunately, the environmental signatures these liposomes rely on are often heterogeneous and dynamic, which leads to unsatisfactory specificity in some circumstances. Therefore, design of delicate liposomes that react to physiological trigger remains a fascinating challenge. Heat, light and ultrasound are common endogenous factors used in these stimuli-responsive strategies. In cancer treatment, photothermal therapy (PTT) and photodynamic therapy (PDT) are two promising strategies in recent years. PTT and PDT are often applied synergistically with surgery and chemotherapy. PTT are commonly realized by materials with high photothermal conversion efficiency that generates heat under near-infrared (NIR) light radiation. In liposomal platform design, PTT can be combined with NIR-triggered API release. Phospholipids undergo phase transition at a certain temperature, by using proper phospholipids such as DPPC, DSPC, the liposome can response to heat generated by NIR light radiation and release the loaded API above transition temperature (Tm) at which the liposome changes from solid phase to liquid phase. PDT therapy are usually realized by photosensitizer accumulated in cancer tissues by liposomal targeted delivery. The photosensitizer interacts with oxygen under light radiation of particular wave length and leads to a series of chemical reaction. During the process, toxic reactive oxygen species (ROS), superoxide oxygen O2 and ·OH are produced, which leads to death of target cancer cells [130, 131]. Haeri and coworkers developed a thermosensitive liposomal system. The thermosensitive liposome (TSL) was further modified with Fab’ fragments of EGFR antibody cetuximab (Fab’-TSL) and EGFR specific peptide GE11 (GE11-TSL). The Fab’-TSL exhibited adequate stability at 37 °C and a temperature dependent release manner when above 40 °C in serum. The result showed that hyperthermia and Fab’-conjugation enhanced the cytotoxicity of DOX-loaded liposome [132]. A delicate gold wrapped immunoliposome was designed to treat breast cancer. The liposome was modified with HER2 antibody and loaded with antitumor medicine cyclopamine. “Petal-like” gold nanocluster was modified on the liposomal surface to provide photothermal conversion under NIR radiation. With the heat provided by NIR radiation and photothermal conversion feature, the thermosensitive liposome shows controlled release ability. The presence of gold nanoparticle also makes multimodal imaging possible. The result showed that the liposome possessed good colloidal stability, NIR-sensitive drug release feature and Photothermal treatment effect [133]. To enhance PDT effect in cancer treatment, a new photosensitizer (BODIPY-I-35) was developed and encapsulated into liposomes, and the liposome was incubated with urea to enhance production of ROS. The result showed that the synergistic effect of liposome and urea provided significantly higher phototoxicity than liposome alone in Hela cells and lowered the dose needed in PDT therapy [134]. In a word, liposome is vastly studied in PTT and PDT therapy and might be promising combined cancer therapy nanoplatform with thermosensitive stimuli-release feature.

Efforts have been made to improve specificity of API-loaded liposomes and reduce off-target effect. Ultrasound (US) and magnetic field are two possible strategies. Acoustic cavitation is the main mechanism of ultrasound-sensitive liposomes. Perfluorocarbon (PFC) is used in these liposomes and causes phase transition from liquid to gas when exposed to US pulses. The liposomal bilayer will be disrupted during the process thus release the encapsulated drugs [135]. Magnetic field can be used to guide magnetic liposomes consisting of iron oxide nanoparticles to target areas. Dwivedi [136] developed a liposome-microbubble conjugate with enhanced delivery in cancer therapies. The liposome can be activated by US and has magnetic targeting ability with citrate-stabilized iron oxide nanoparticles. The in vitro studies showed that the liposomal platform promoted apoptosis under US pulses, and the liposome was highly effective in killing pancreatic cancer cells at a low dose. Liposomes with ultrasound triggered release feature has been extensively studied in cancer therapy and could be a promising approach to improve drug delivery specificity [137–139].

Based on heterogeneous characteristics of TME and nanomaterials, various stimuli-responsive liposomes are developed. These novel liposomal nanoplatforms exhibit good bioavailability, prolonged drug half-life, improved specificity in cancerous areas and synergistic effect with various APIs. However, performance of these liposomes has merely been tested in cell lines and mouses, and human body is an extremely complex system and requires further research to achieve concrete drug efficacy and toxicity information with the corresponding stimuli needed.

## Recent advances of common LBDDS application in nucleic acid delivery

Lipid-based drug delivery systems (LBDDSs) have been vastly researched in nucleic acid delivery. Nucleic acids are being used to develop vaccines, treat cancer and other diseases caused by gene defects such as cystic fibrosis, muscular atrophy, hemophilia and sickle cell disease [140–145]. In cancer gene therapy, siRNA can induce degradation of specific messenger RNA (mRNA) and is commonly used to silence expression of specific genes. And cancer immunotherapy uses mRNA as ingredients for vaccines. Modified virus is an option for effective gene delivery. However, application of viral vectors is limited due to the immunogenicity, potential oncogenicity and relatively small capacity [146]. LBDDS exhibit less immunogenicity and mutagenesis compared with viral vectors. Liposome and LNP are two common vehicles for nucleic acid delivery. As siRNA delivery requires high specificity towards cancer cells, immunoliposomes with active targeting ability or stimuli-responsive liposomes are vastly studies in vivo and in vitro [110, 147–149]. Liposomes are also used to fabricate mRNA tumor vaccines [150]. Since nucleic acids are negatively charged, cationic liposomes were used to effectively deliver nucleic acids initially [151]. 1,2-di-O-octadecenyl-3-trimethylammonium propane (DOTMA) and 1,2-dioleoyl-3-trimethylammonium-propane (DOTAP) are two commonly used cationic lipids [152]. However, cationic liposomes are easily purged by the mononuclear phagocyte system (MPS) which consists of cells in liver and spleen. Cationic charges induce cytotoxicity and are more likely to stimulate the immune system and cause aggregation in blood [153].

As a new generation LBDDS, LNP has shown impressive delivery efficacy and bioavailability in nucleic acid delivery. Similar to liposomes, LNPs is composed of lipids, phospholipids, cholesterol and PEG. These materials form 80–200 nm nanoparticles and deliver nucleic acids into the cells through endocytosis [154]. LNP is broadly explored in siRNA and mRNA delivery. Ionizable lipids are the main lipids used in LNPs. These lipids are neutral at physiological pH and protonated at low pH. LNPs with neutral lipids have less interactions with anionic membranes of blood cells and exhibit better biocompatibility and less cytotoxicity than cationic liposomes [155]. LNPs are used to deliver siRNA and mRNA. siRNA is able to silence expression of target proteins in liver targets, and researches show that the adsorb apolipoprotein E (ApoE) expressed mainly on livers is important in the process [156]. Mennati and coworkers manufactured a hybrid LNP loaded with siRNA and lycopene. The siRNA was used to silence insulin-like growth factor-1 receptor (IGF-1R), and researches showed that blockage of IGF-1R signal reduces cell proliferation and leads to programmed cell death. Lycopene is a chemical with anti-tumor effect. The result showed that the LNP significantly induced apoptosis and arrested cell cycle in breast cancer MCF-7 cell line [157]. siRNA is also used to knock down long non-coding RNAs (lncRNAs) selectively expressed with oncogenic functions. A delicate LNP nanocarrier was developed loaded with novel oncogenic lncRNA LINC01257 to treat children with acute myeloid leukemia (AML). The characterization of the LNP was carefully designed and measured and researchers verified that the LNP-siRNAs were taken up by cancer cells. The result showed that the expression of LINC01257 was significantly reduced [158]. However, delivery of these siRNAs in cancer gene therapy still faces off-target and potential safe issue and demands further research.

**Fig. 4:**
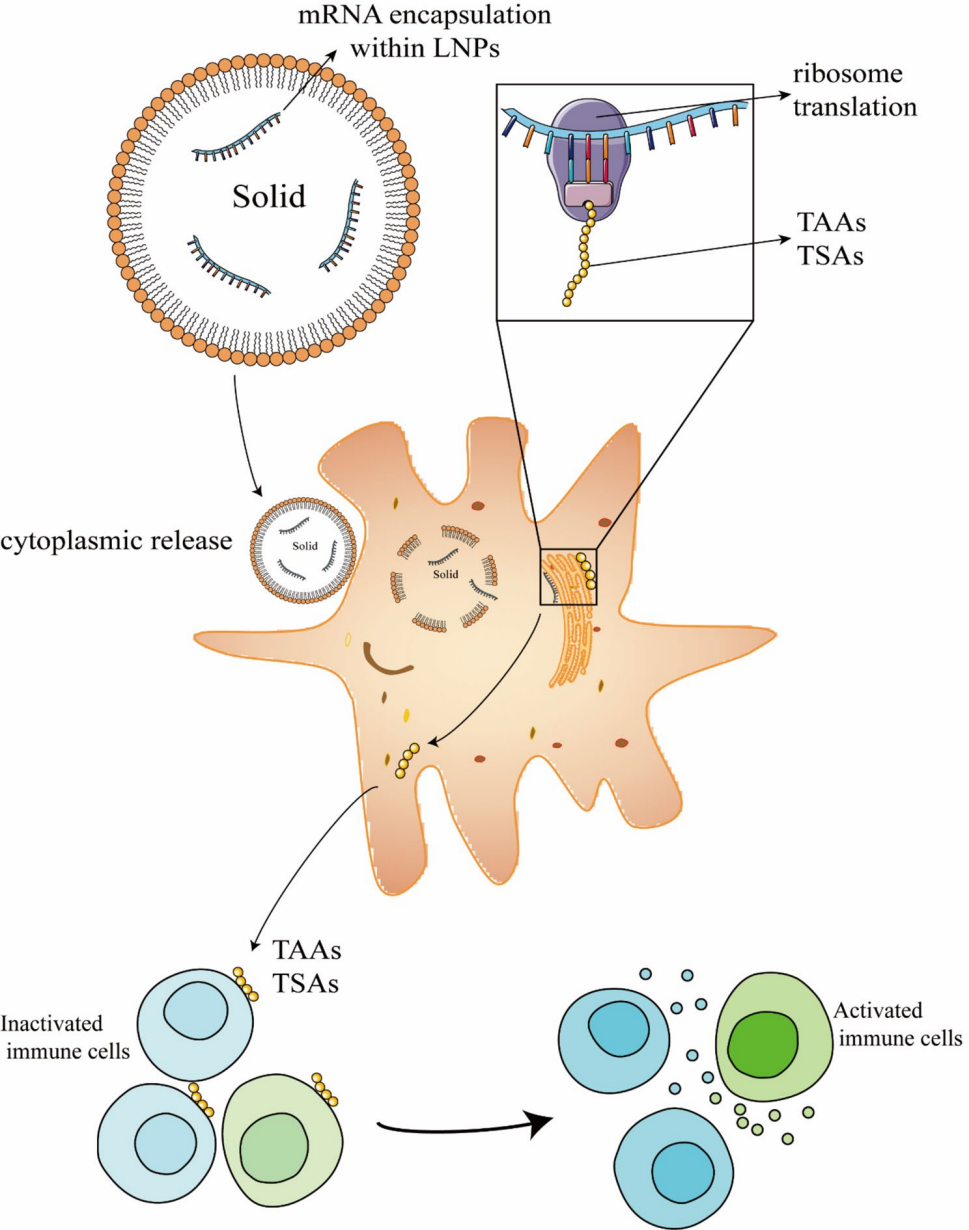
LNP based mRNA vaccines induce tumor vaccination. mRNAs are encapsuled in the LNP nanocarrier and released in the cytoplasm, TAAs and TSAs are synthesized by the ribosome and released from the cytoplasm. TAAs and TSAs activate the immune cells and eventually induce tumor vaccination

For mRNA delivery, LNP is the most advanced nano-platform. Exogenous mRNAs lead to expression of the target protein in the desired cells and stimulate specific immune response, and generate antibodies that achieve vaccine effects. mRNA vaccine for COVID-19 has been put into emergency use and LNP is the key carrier in the system. Cancer immunotherapy includes immune checkpoint blockade, chimeric antigen receptor (CAR)-T cell therapy, immune system modulator and cancer vaccine [159]. These therapies all involve function of immune system and mRNA loaded LNP is a promising strategy of cancer vaccines. mRNAs used in these LNP vaccines mainly encode tumor associated proteins (TAAs) and tumor specific antigens (TSAs). By encapsulating these mRNAs into carefully designed LNPs, degradation of mRNA is reduced and mRNA can pass through the cell membrane by endocytosis. The process of tumor vaccination induced by mRNA loaded LNP is shown in Fig. 4. A clinical trial was conducted to induce therapeutic vaccinations in gastrointestinal cancer. Antigen-related genes and mutations of driver gene were concatenated into a mRNA and the mRNA was loaded into a LNP and used to vaccinate metastatic gastrointestinal cancer patients. The result showed that the vaccine was safe and corresponding mutation-specific T cell responses were elicited [160]. Zhang et al. developed a mRNA vaccine with lipid-like material. A special LNP that could activate Toll-like receptor 4 (TLR4) and induce T cell activation was designed and screened. The result showed that the C1-mRNA vaccine delivered mRNA to DCs and promoted antigen presentation. The LNP also exhibited self-adjuvant property [161]. Despite these inspiring advances in LNP-based mRNA vaccines, the targeting ability of LNPs needs to be further researched. Intravenously (IV) administered LNPs tend to accumulate in the liver [162], and strategies have been utilized to break this limit. LoPresti reported that by replacing helper lipids with charged alternatives, the nanoparticle can be targeted to the spleen and lungs. In the experiment, DOPE was replaced by neutral lipids, anionic lipids and cationic lipids. The result exhibited a method to target LNPs at the spleen or lungs [163]. A special LNP with selective organ targeting (SORT) component was developed and conjugated to LNP surface. CRISPR-Cas is a power tool to edit genes. However, the off-target effect limited the application of CRISPR-cas. A research showed that by encapsuling CRISPR-Cas system into the LNP system, the nanocarrier can distribute specifically in target cancer tissues. By adding specific antibodies, the nanocarrier reduced off- targeting and enhanced the targeting ability [164]. By adding SORT molecule, the LNP can target at spleen, lungs and other organs [165]. This shows that compared with plain LNPs, the coated SORT molecule could greatly enhance the targeting ability of LNPs.These research on mRNA vaccines showed that nucleic acid delivery through LNPs is a promising way in fabricating tumor vaccines as well as cancer immunotherapy.

In addition to liposomes and LNPs, other LBDDSs including nanoemulsions, nanosuspensions and niosomes have also been explored to deliver nucleic acids, and these lipid nanomaterials exhibit unique advantages. These materials are also researched to be used in cancer vaccines. Table 4 shows examples of these materials that are applied in cancer treatment and cancer vaccines. When developing nucleic acid loaded LBDDSs, the ethical issue and potential socio-economic impacts should also be evaluated. As nucleic acid vaccines directly interfere with gene transcription and translation, patients’ informed consent, autonomy and privacy should be well taken care of. Moreover, the potential impact of gene vaccines on human body should be well monitored and evaluated [166, 167]. The relatively high price of lipid-based gene vaccines could result in injustice in terms of cancer immune [168, 169]. These issues should be constantly evaluated during vaccine development and government policy formulation.

**Table 4:** Examples of LBDDS applied in cancer treatment and cancer vaccines

Lipid nanocarrier	Pharmaceutical ingredients	Applications	Outcome	References
Liposome	mRNA encoding cytokeratin 19	Lung cancer vaccine	The vaccine provoked a strong cellular immune response and tumor growth was slowed in an aggressive Lewis lung cancer model.	[150]
LNP	siRNA and lycopene	Breast cancer treatment	The LNP induced apoptosis and arrested cell cycle in the MCF-7 tumor cell lines.	[157]
LNP	mRNA	Gastrointestinal cancer vaccine	The vaccine was safe and elicited mutation-specific T cell responses against predicted neoepitopes not detected before vaccination.	[160]
Nanoemulsion	TLR7/8 agonist (R848)	Cancer vaccine	NE (R848) induced tumor-specific T cell activation and mitigating T cell exhaustion and showed systemic antitumor immune responses in mouse lung cancer models.	[170]
Nanosuspensions	Glioma cell membrane and DTX	Anti-glioma therapy	The nanosuspension exhibited effective glioma cell targeting ability and induced efficient antigen presentation and downstream immune.	[38]
Niosome	Artemether	Breast cancer treatment	The artemether-loaded noisome caused superior tumor necrosis and smaller tumor volume compared to artemether group.	[171]

## Limitations and challenges

### Protein corona on LBDDSs

Despite vast number of researches on common LBDDSs including liposomes and LNPs in cancer treatment, there are still concerning issues in clinical translation. APIs of liposomal drugs that are applied in clinical use are mostly chemical drugs, and these liposomes are barely decorated with active targeting ligands. Research show that the ligands used to modify the system may lead to formation of protein corona (PC). When the LBDDS is absorbed to blood circulations through various methods, the nanocarrier interacts with blood cells and plasma proteins and forms a PC with an average thickness of 50–80 nm on liposomal surface [172, 173]. After entering the biological blood circulation, the nanocarrier is instantly coated by proteins with high abundance and low affinity in blood and forms the “soft corona”. With the residence time increases, the “hard corona” composed of proteins with high affinity is formed on the outer surface [174]. The formation of PC is considered a consequence of minimization of free enthalpy, and it is also related to van der Waals forces, hydrogen bonding and hydrophobic interactions [175].

By changing the interacting surface of the LBDDS, PC affects properties including circulation and retention time in the system, distribution and release of encapsulated drugs, cell uptake and clearance [176]. Moreover, formation of PC in liposomal surface alters zeta potential of the system, and the proteins could contribute to solubilization of the lipids therefore disrupt the phospholipid bilayer. Adsorption of proteins may also lead to aggregation [177]. For active targeted liposomes, the molecules and ligands on the surface induce more potential binding of plasma proteins compared with passive targeted liposomes. However, whether formation of PC enhances macrophage function still needs further research [178]. The effect of PC on lipid nanoplatform internalization is associated with liposome composition and the bioenvironment of the nanoplatform [179–181].

The pharmacokinetic influence of PC has been broadly researched in recent years. Prolonged circulation enhances the therapeutic effect of the nano-drug. Proteins constituting the PC can be divided into two types, which are opsonins and dysopsonins [182]. Opsonins include immunoglobulins and complement proteins, and these proteins enhance MPS capture of the nanocarrier. On the other hand, dysopsonins such as albumin and apolipoproteins can promote the circulation period [183]. For active targeting strategies, PC can also cover the ligand binding site potentially therefore leads to reduction of targeting efficacy. For instance, folate receptor is overexpressed in glioma cells and folic acid (FA) was modified on some liposomal nanocarriers. Due to undesired adsorption of plasma proteins especially natural IgM, the FA modified liposomes mainly accumulate in microphages, and the liposome tend to accumulate more in liver and spleen instead of tumor cells [184, 185].

Two approaches have been explored to reduce the formation of PC formation and minimize unintended protein adsorption. The first approach is reducing PC formation on LBDDS surface. PEG modification on liposomes have been proved to form a hydrated layer and reduce protein adsorption. By stealth coating, the PEGylation greatly reduces protein adsorption in blood circulation, therefore improves the pharmacokinetics and reduces immunogenicity [186, 187]. However, conventional PEG induces anti-PEG antibody and decreases long circulation ability of the nanocarrier. The Fc domain of anti-PEG antibody activates endocytosis and complement effect, leading to increased blood clearance of nanocarriers and accumulation in liver and spleen. The immunoreaction can also affect the nanoplatform structure [188–190]. Researches have been conducted to overcome these drawbacks. It was reported that an anti-PEG single-chain variable fragment (PEG-scFv) was coated onto liposomes. Compared with full PEG ligand, the PEG-scFv exhibited reduced binding with anti-PEG IgM while preserving the PEG stealth coating function in rats with pre-existing anti-PEG antibodies [191]. Another typical stealth material is the zwitterionic polymer. Characterized by electrostatic interactions with water molecules, the zwitterionic polymer can form a hydrated layer that reduces nonspecific adsorption [192, 193].

To minimize the unfavored result of PC formation, researches have also been conducted to modify the PC constitution. Kim and coworkers designed a siRNA carrier coated with polydopamine, and proteomics analysis identified that the main PC constitution was albumin. Albumin mediated nanocarrier endocytosis and the nanocarrier significantly attenuated CT26 tumor growth [194]. Research shows that apolipoproteins promote nanocarrier delivery to the brain through low density lipoprotein receptor (LDLR). Surface polysorbate modification causes apolipoprotein adsorption therefore improves delivery to the brain [195].

The protein corona formed on the LBDDS affects the biological characteristics. To better predict PC formation, artificial intelligence (AI) has been used in analyzing the interaction between the nanocarrier and PC. AI technology main includes machine learning (ML), deep learning (DL) and artificial neural networks (ANN) [196, 197]. These promising technologies showed great potential in nano-platform design, early cancer diagnosis and analyzing drug distribution and efficacy. Since the PC is mainly composed of various plasma proteins, it is extremely hard to test the composition in vivo [198]. Compared with in vivo studies, nanocarriers tend to adsorb less proteins in the surface due to the monotonous cell culture environment [174].Based on reported documents of protein corona, AI technology can predict possible PC elements and endosomatic biological effect. Traditional linear regression model showed unfavorable prediction ability with low *R*^*2*^ (less than 0.40). By applying a machine learning model and meta-analysis, Ban et al. constructed a model that precisely predicts the functional protein compositions including immune proteins, complement proteins and apolipoproteins [199]. Another research applied a ferritin nanocage loaded with drugs and tracers to various tumor models, and the nanoparticle permeability in cancerous vasculatures were predicted by image segmentation-based machine learning techniques. This research refined the design of the nano-drug and improved the drug permeability to solid tumors [200].

Recent studies also reveal that change of incubation environment from 90% fetal bovine serum to 82% murine plasma significantly alters the test result of targeting capability that associates with PC formation. This suggests that to study the effect of PC formation in liposomal platforms, the experimental setting should be as close as possible to the actual environment. PC formation is an important issue hindering clinical translation of LBDDS and requires further research. AI is a powerful tool in PC prediction and nano-platform design improvement. Focusing on specially modified LBDDS surface design to either reduce PC formation or induce specific protein adsorption to refine nano-drug pharmacokinetics and immunogenicity are also promising mitigation strategies.

### Obstacles in clinical translation

Other major obstacles in the clinical translation of LBDDSs lies in large-scale manufacturing, government regulations and intellectual property issues. Although various active targeting and stimuli-responsive strategy have been broadly studied, the currently approved LBDDSs are all designed with passive targeting strategies. In other words, clinical translation of liposomes and LNPs based on active targeting and stimuli-release was hindered by certain reasons. Quality control and cost control are two major issues when it comes to clinical translation. Compared with plain liposomes, liposomes and LNPs with specific ligands that lead to designed immunoreaction faces more difficulties in quality control. More procedures are required to integrate these components into the nano-platform, making it more difficult to maintain uniform nano-drug characteristics [201, 202]. And evaluating the pharmacokinetics, toxicity and pharmacodynamics becomes more complicated. PC formed on these nanocarriers are also more complicated than plain liposomes [87, 141]. Cost of the production procedure also increases as the nanocarrier becomes more complicated. Different from laboratory design, massive production in factories of lipid-based nanomaterials always faces plenty of challenges. With more extra modifications and moieties, the fabrication and storage cost increase. The government regulations make a difference in LBDDS clinical translation. The intellectual property also influences the clinical translation of LBDDSs, as the current intellectual property restricts the knowledge share of LBDDSs and may increase the cost of LNP design and manufacture. To overcome these obstacles, reports of relative studies should be as detailed as possible to increase the repetition rate of the experiments. More open policies of LBDDS regulations and intellectual property should be proposed after concrete consultation with academia, industry and law. These measures will speed up the clinical translation of LBDDSs.

liposomal clinical use is lack of authoritative criteria that can evaluate the efficacy and safety of LBDDS universally. Especially for active targeted and stimuli-responsive nano systems. In the active targeting strategy, the ideal scenario that the delicate moiety placed on the liposomal surface guide the system towards cancerous areas is impeded by PC formation. Since the incubation environment affects the evaluation of targeting capability, a standard procedure is needed to make the test result demonstrate the actual situation in human body. Proteomics-based mass spectrometry (MS) is a powerful tool to examine the PC component and could be made a standard procedure in active targeted lipid-based nanocarrier design. Similar to these concerns, aggregation of liposomes can be resulted from proportion of each component, surface charge and density of ligands decorated on the surface. Test of the aggregation should also be carried out with a unified criterion. Compared with traditional chemotherapy and passive targeted liposomes, off-target effect and consequent toxicity towards normal cells caused by active-targeted liposomes are also concerning obstacles between lab and clinic use. Caracciolo and coworkers found out that there is significant difference of liposome PC in mice and humans [203], indicating tests of active targeted liposomes should not only be tested in cell lines and mice, but also in organoid or mammal models. Safety issue is also a limitation of many gene-delivery lipid carriers. Tumor gene therapies use nucleic acid as API and aim to express or inhibit expression of specific proteins in target cells, the off-target effect and potential concern towards gene transfection are important issues that limit the clinical translation of these drug platforms. To overcome this limitation, further researches on active targeting LBDDS should be conducted.

## Perspectives

Over the years, liposomes, LNPs and other lipid-based nanocarriers have become a powerful weapon against cancer. Various chemical drug-encapsulated liposomes have been applied to clinical use. Liposomes and LNPs possess ideal bioavailability and biocompatibility and few toxicities. Tons of studies proved that by using these materials, therapeutic drugs can be specifically delivered to tumor sites with prolonged half-life, deep penetration and effective cellular internalization. However, further work should be carried out to establish standard effectiveness evaluation criteria in active targeted lipid nanocarriers. Mass spectrometry techniques can be utilized to studies the composition and formation of protein corona and eventually enhance the active target ability.

## Conclusion

Lipid-based drug delivery systems are promising anti-tumor drug carriers. To achieve better clinical translation and more promising therapeutic results, future research can be conducted focusing on active targeting strategies and overcoming off-target effects. By refining the combination of PEG with active targeting moieties and improving stimuli-release approach based on the biological characteristics of the cancerous tissue, the targeting efficacy can be elevated. AI and machine learning can also be used to improve LBDDS design and reduce unfavorable influence of PC formation, therefore overcoming off-target effects. and it is hoped that these nanocarriers will be used clinically in near future with more promising therapeutic results.

## Data Availability

No datasets were generated or analysed during the current study.

## Abbreviations

ANN: Artificial neural networks
AI: Artificial intelligence
COVID-19: Coronavirus disease 2019
CHEMS: Cholesteryl hemisuccinate
DAPE: Diacetylenic-phosphatidyl-ethanolamine
DL: Deep learning
DOPE: Dioleoyl-phosphatidyl-ethanolamine
LNP: Lipid nanoparticles
ML: Machine learning
MDR: Chemotherapy can also cause multi-drug resistance
NLC: Nanostructured lipid carriers
POPE: Palmitoyl-oleoyl-phosphatidyl ethanolamine
PHC: N-palmitoyl-l-homocysteine
LBDDS: Lipid-based drug delivery system
SUV: Small unilamellar vesicles
LUV: Large unilamellar vesicles
MLV: Multilamellar vesicles
MVL: Multivesicular liposomes
NP: Nanoparticle
MPS: Mononuclear phagocyte system
PEG: Polyethylene glycol
FDA: Food and Drug Administration
EPR: Enhanced permeability and retention
TME: Tumor microenvironment
TfR: Transferrin receptor
FR: Folate receptor
ER: Estrogen receptor
BBB: Blood-brain barrier
GBM: Glioblastoma multiforme
Dox: Doxorubicin
DTX: Docetaxel
NSCLC: Non-small cell lung cancer
CD44: Cluster of differentiation 44
HA: Hyaluronic acid; CS: Chondroitin sulfate
EGFR: Epidermal growth factor receptor
EGF: Epidermal growth factor
AgNPs: Silver nanoparticles
HER2: Human epidermal growth factor receptor 2
ECM: Extracellular matrix
PD-L1: Programmed cell death ligand 1
ICI: Immunocheckpoint inhibitor
VEGF: Vascular endothelial growth factor
VEGFR-2: Vascular endothelial growth factor receptor-2
ssDNA: Short single-stranded DNA
ssRNA: Single-stranded RNA
SELEX: Systematic evolution of ligands by exponential enrichment
API: Active pharmaceutical ingredient
PE: Phosphatidylethanolamine
MMP: Matrix metalloproteinase
TLF-9: Toll-like receptor 9
ATO: Arsenic trioxide
PTT: Photothermal therapy
PDT: Photodynamic therapy
NIR: Near-infrared
ROS: Reactive oxygen species
TSL: Thermosensitive liposome
US: Ultrasound
PFC: Perfluorocarbon
MPS: Mononuclear phagocyte system
IGF-1R: Insulin-like growth factor-1 receptor
AML: Acute myeloid leukemia
TAAs: Tumor associated proteins
TSAs: Tumor specific antigens
TLR4: Toll-like receptor 4
SORT: Selective organ targeting
PC: Protein corona
MS: Mass spectrometry
